# The timing of administration of intravenous dexmedetomidine during lower limb surgery: a randomized controlled trial

**DOI:** 10.1186/s12871-016-0282-2

**Published:** 2016-11-21

**Authors:** Eunsu Kang, Ki Hwa Lee, Sang Yoon Jeon, Kyu Won Lee, Myoung Jin Ko, Hyojoong Kim, Yong Han Kim, Jae-Wook Jung

**Affiliations:** 1 Department of Anesthesiology and Pain Medicine, Haeundae Paik Hospital, Inje University 1435, Jwa-dong, Haeundae-gu, Busan, 612-862 Korea; 2 Department of Anesthesiology and Pain Medicine, Cheju Halla General Hospital, 65, Doryeong-ro, Jeju-si, Jeju-do, 63127 Korea

**Keywords:** Dexmedetomidine, Hypotension, Spinal anesthesia

## Abstract

**Background:**

Dexmedetomidine, a selective alpha-2 agonist, has sedative, analgesic, and anxiolytic effects without respiratory depression. Dexmedetomidine can cause a biphasic cardiovascular response, and induce transient hypertension. Hypotension is a common complication of spinal anesthesia. Decreasing anxiety of patients before procedure is important for high quality of procedure. This study aimed to compare the incidence of hypotension and patients’ anxiety and comfort levels when dexmedetomidine was intravenously administered before and after spinal anesthesia.

**Methods:**

Seventy-four patients with American Society of Anesthesiologists physical status classification I or II were randomly allocated into two groups. Spinal anesthesia was performed using 12 mg of 0.5% heavy bupivacaine. In Group A, 1 μg/kg of dexmedetomidine was intravenously administered for 10 min, followed by the maintenance infusion of dexmedetomidine 0.2 μg/kg/hr after 5 min of intrathecal bupivacaine injection. Patients in Group B received same dose of dexmedetomidine by intravenous administration before 5 min of intrathecal bupivacaine injection. Perioperative vital signs, anxiety (using the Spielberger’s State-Trait Anxiety Inventory) and comfort (using the numerical rating scale) were evaluated.

**Results:**

The incidence of hypotension was significantly lower in Group A (16.1%) than in Group B (48.4%) during infusion of dexmedetomidine (*p* = 0.01). The need for treatment of hypotension is higher in Group B than Group A (*p* = 0.02). The incidence of bradycardia and desaturation did not significantly differ between the two groups. There were no statistically significant differences regarding the patients’ anxiety and comfort.

**Conclusions:**

Hypotension is more frequently occurred, and the treatment of hypotension is more needed in Group B. The intravenously administration of dexmedetomidine before spinal anesthesia has no advantages in hemodynamic status and patients’ comfort compared to that after spinal anesthesia during lower limb surgery.

**Trial registration:**

ClinicalTrials.gov number, NCT02155010. Retrospectively registered on May 22, 2014.

## Background

Spinal anesthesia (SA) is commonly used for lower limb surgery because it has a high success rate and can maintain spontaneous ventilation. The hemodynamic instability such as hypotension and bradycardia are the most common side effects with SA [1, 2]. It may be increased when sedatives is used with SA for decreasing patients’ anxiety.

Patients want to be sedated during painful procedures such as spinal tapping. Pre-sedation of patients can be an important factor in the quality of SA. Propofol, midazolam, and dexmedetomidine (DEX) have been commonly used for sedation during SA. Propofol is a profound respiratory depressant and midazolam has a slight synergistic ventilatory depressive effect with SA [3]. DEX, a selective α_2_-adrenoceptor agonist, causes minimal or no respiratory depression, unlike other sedatives [4].

Intravenous DEX as a rapid infusion caused biphasic changes in heart rate (HR) and mean blood pressure (MBP) [5]. Dyck et al. [5] found a 22% rise in the MBP and a 27% decline in the HR within 4-5 min after starting intravenous infusion of DEX. Although they infused DEX at a high dose (2 μg/kg for 5 min), the administration of a bolus of 1 μg/kg DEX initially causes an increase in BP for a brief period [6].

Also, DEX has anxiolytic effects and there is growing interest in comparing efficacy, time to onset, and outcomes between DEX and other anxiolytics such as midazolam [7]. DEX was as effective as higher dose (0.06 mg/kg) of midazolam in preoperative sedation [8]. Anxiety-like behavior is reduced by decreases in the circulating catecholamine secretion after the administration of DEX [9].

Therefore, we evaluated the hemodynamic effects and patients’ anxiety and comfort level according to the timing of administration of DEX with SA.

## Methods

### Study design and patient population

This randomized, double-blind study enrolled seventy-four patients from March, 2014 to July, 2015. This prospective study was approved by the Institutional Review Board of Inje University Haeundae Paik hospital (129792-2014-003), and written informed consent was obtained from patients (ClinicalTrials.gov number, NCT02155010). This study protocol complied with the 1975 Declaration of Helsinki.

### Criteria for inclusion and exclusion

Patients who were classified as American Society of Anesthesiologists physical status I or II, aged 20-60 years, and scheduled to undergo lower limb surgery with SA (expected surgical time <90 min) were included. Patients with hypertension, diabetes mellitus (DM), or heart disease (bradycardia or atrioventricular block) were excluded.

### Preoperative preparations and anesthesia protocol

No premedication was administered to the patients. While in the operating room, all patients were monitored by electrocardiography (ECG), pulse oximetry, non-invasive blood pressure (NIBP), and bispectral index (BIS) values. Before SA, crystalloid 6 mL/kg was intravenously injected to the patients for hydration. A nurse who did not participate in this study prepared a 50 mL mixture of normal saline 48 mL and DEX (Precedex®, Hospira Inc., Rocky Mount, NC, USA) 200 μg. An anesthesiologist who did not participate in this study performed SA. Patients were randomly assigned to Group A and B (*n* = 37, each group) (www.random.org). Patients were intravenously administered 1 μg/kg of DEX for 10 min, followed by the maintenance infusion of DEX 0.2 μg/kg/hr. Group A and B were administered DEX after and before 5 min of spinal anesthesia. Patients were placed in the lateral decubitus position and a spinal tap in the L3/4 or L4/5 intervertebral space was performed using the midline approach. After confirming the free flow of the cerebrospinal fluid, 0.5% heavy bupivacaine (Marcaine®, AstraZeneca AB, Södertälje, Sweden) 12 mg was injected intrathecally. We evaluated the disappearance of pinprick sensation by using a needle in the mid-clavicular line. In addition, we confirmed the extent of motor blockade by using the modified Bromage scale [10]. The administration of DEX was discontinued when the surgeon began to suture. Following arrival in the PACU, patients stayed for one hour. The patients were discharged from the PACU when the modified Aldrete score was ≥ 9.

### Measurements

MBP, HR, BIS values, and oxygen saturation were recorded at baseline and at 5 min intervals during DEX infusion and in the post-anesthetic care unit (PACU). The primary outcome was comparison of the incidence of hypotension between the two groups. Hypotension (defined by a decrease in MBP below 20% of baseline), bradycardia (HR < 50 beats per min [bpm]), and oxygen desaturation (SpO2 < 95%) were recorded. Ephedrine 5 mg or atropine 0.5 mg was administered intravenously when hypotension or bradycardia occurred. O_2_ 3 L was applied via nasal cannula when oxygen desaturation occurred. The secondary outcome was comparison of patients’ anxiety and comfort. The patients’ levels of anxiety experienced before (preoperative) and after (discharge from PACU and postoperative 1^st^ day) the study was assessed using the Spielberger’s State-Trait Anxiety Inventory (STAI) [11]. Patients’ comfort during (10 minutes after SA) and after (discharge from PACU) this study was assessed using the numerical rating scale (NRS, 0 = not as comfortable as imaginable, 10 = very comfortable). All data were recorded by another anesthesiologist who did not know when DEX was injected intravenously.

### Statistical analysis

A sample size calculation was based on a pilot study performed with 6 cases in each group. In the pilot study, incidence of hypotension in Group A was 33% less than in Group B. The requires number of patients for each group was thirty-three (α =0.05, β =0.8). Assuming a drop-out rate of 10%, the final sample size was set at thirty-seven patients per group. The data are expressed as mean ± standard deviation (SD), median (interquartile range, IQR) or numbers of patients. Statistical analyses were performed using repeated-measured analysis of variance, the chi-squared test, the independent t test, and the Mann-Whitney test. All statistical analyses were performed using SPSS version 21.0 (SPSS Inc., Chicago, IL, USA) and Medcalc 14.12.0 (MedCalc Software bvba, Ostend, Belgium). A p-value < 0.05 was considered to indicate statistical significance.

## Results

**Fig 1:**
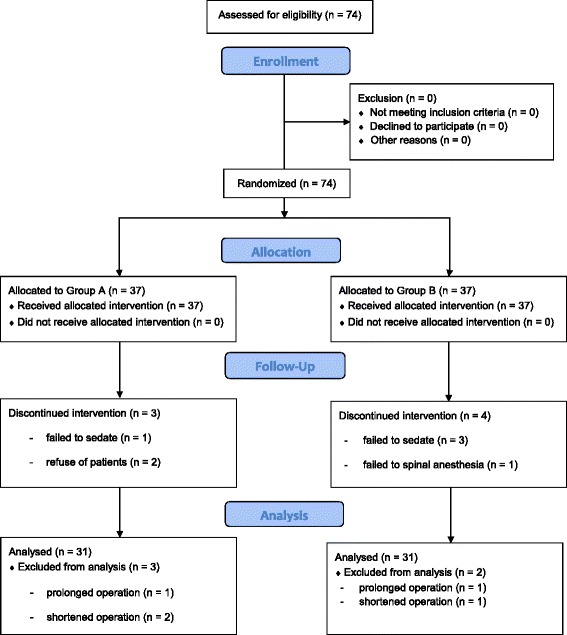
CONSORT flow diagram

A total of seventy-four patients were enrolled in this study, but six patients per group were excluded from data analysis. The final analyses included thirty-one patients per group (Fig. 1).

Patients’ baseline characteristics were not different between the two groups (Table 1).

**Table 1:** Patients’ baseline characteristics

	Group A (*n* = 31)	Group B (*n* = 31)	*P* value
Sex (male/female)	22/9	15/16	0.12
Age	35.87 ± 11.22	38.74 ± 12.27	0.34
ASA (1/2)	26/5	26/5	0.70
Height	170.25 ± 6.14	167.22 ± 9.34	0.14
Weight	71.22 ± 10.02	67.62 ± 12.10	0.21
Infusion time of DEX (min)	86.93 ± 17.78	86.61 ± 12.99	0.94
Injection site (L3-4/L4-5)	13/18	15/16	0.80
Maximum sensory block level	T10 (8-10)	T10 (8.5-10)	0.16

Values are expressed as number of patients, mean ± SD, and median (IQR). *ASA* American society of anesthesiologists classification, *DEX* dexmedetomidine, *L* lumbar, *T* thoracic dermatome, *SD* standard deviation, Injection site, intrathecal heavy bupivacaine injection site, *IQR* interquartile range

The incidence of hypotension was significantly lower in Group A (16.1%) than in Group B (48.4%) during infusion of dexmedetomidine. The incidence of bradycardia and desaturation did not significantly differ between the two groups (Table 2). Also, the BIS values were not significantly different between two groups during infusion of DEX (Group A vs. Group B, 72.39 ± 8.96 vs. 71.16 ± 10.46).

**Table 2:** Hemodynamic variables during infusion of dexmedetomidine

	Group A (*n* = 31)	Group B (*n* = 31)	*P* value
Desaturation (Yes/No)	9/22	6/25	0.55
Bradycardia (Yes/No)	11/20	9/22	0.79
Hypotension (Yes/No)	5/26	15/16	0.01^*^
Frequency of injection of ephedrine (0/1/2/3/4)	29/1/1/0/0	17/9/2/2/1	0.02^*^

Values are expressed as number of patients

The MBP and HR in the PACU were did not statistically differ between the two groups. The mean HR (Group A vs. Group B, 57.88 ± 6.16 vs. 59.49 ± 8.13, *p* = 0.133) and MBP (Group A vs. Group B, 80.07 ± 12.48 vs. 77.49 ± 9.36, *p* = 0.121) were not significantly different. Desaturation did not occurred in the PACU in either group. The BIS values of Group A and B were 88.94 ± 5.19 and 88.09 ± 5.96 in the PACU, respectively (*p* = 0.54).

The preoperative State Anxiety Inventory scale and Trait Anxeity Inventory scale showed significant correlation (*r* = 0.59, *p* < 0.0001). The State Anxiety Inventory scale was decreased during the 3 assessment time periods (*p* = 0.000), but this change was not significantly different between the two groups (*p* = 0.66) (Table 3).

**Table 3:** The Spielberger’s State Anxiety Inventory scale before and after surgery

	SAI (preoperative)	SAI (discharge from PACU)	SAI (postoperative first day)	*P* value
Group A	45.52 ± 12.01	38.55 ± 8.04	34.55 ± 8.64	<0.001
Group B	43.65 ± 14.24	37.03 ± 10.51	34.90 ± 9.03	<0.001

Values are expressed as mean ± SD. *SAI* Spielberger’s State Anxiety Inventory scale, *PACU* post-anesthetic care unit, *SD* standard deviation. The SAI was decreased over time (p = 0.000), but this change was not significantly different between the two groups (*p* = 0.66)

The median (IQR) comfort scores (NRS) of Group A and B during infusion of DEX were 7 (5-9) and 8 (5-9), respectively (*p* = 0.35). Also, the median (IQR) comfort scores upon discharge from the PACU of Group A and B were 7 (5-8.75) and 7 (5-8.75), respectively (*p* = 0.62).

There were no neurologic complications such as paralysis or paresthesia.

## Discussion

Infusion of DEX before SA can increase the incidence rate of hypotension. Although perioperative anxiety was decreased over time, there was no significant difference in patients’ anxiety and comfort level according to the timing of administration of DEX.

DEX has both analgesic and sedative properties that may prolong the duration of SA [12, 13]. The intravenous administration of DEX as premedication with SA can be used widely without serious complications. Lee et al. [14] and Park et al. [15] demonstrated that 0.5 or 1.0 μg/kg of DEX administered as an isolated bolus in the absence of maintenance infusions prolonged the duration of SA, and the incidence of hypotension was found to be 15% and 0%, respectively. We injected an intrathecal dose of 12 mg of heavy bupivacaine to the patients. Although heavy bupivacaine 15 mg was intrathecally injected in another study [16], the incidence of hypotension (0%-16%) was lower than that observed in our study (16.1% and 48.4%). This might be attributed to the single bolus administration of a low dose of DEX (0.5 μg/kg).

A loading dose (1 μg/kg/10 min) of DEX was sufficient for surgery of less than 60 min, and DEX infusion followed by a maintenance dose (0.2 μg/kg/hr) was sufficient for surgery within 90 min without delayed recovery [17]. Thus, we used 1 μg/kg for 10 min for the loading dose of DEX, followed by 0.2 μg/kg/hr for the maintenance dose.

Early vasoconstrictive effects of DEX can induce hypertension, but it could not offset the hypotensive effect of SA. Vasoconstrictive effects seem to be a transient phenomenon and cannot offset the vasodilation due to SA. Rather, it seems that the hypotensive effect was amplified by the SA and DEX in Group B. Also, stimulation of the presynaptic α_2_-adrenoceptor by DEX causes a dose-dependent decrease in the concentration of norepinephrine in plasma and can reduce norepinephrine release by up to 92% in young healthy volunteers [18]. So, we thought that hypotension was more frequently occurred in Group B.

DEX premedication reduced the sympathoadrenal response, analgesic requirements, and anxiety in intravenous regional anesthesia [19]. In addition, DEX may have preventive effects against anxiety-like behaviors and cognitive dysfunctions in rats with post-traumatic stress disorder (PTSD) after repeated administration [9]. Hyperactivity of the noradrenergic systems is thought to be a feature of PTSD and the attenuation of noradrenergic activation by clonidine has been found to ameliorate symptoms of PTSD [20]. Patients’ mean anxiety scores (using the Anxiety Assessment Scale) were significantly lower in the DEX group than in the placebo group after MAC (monitored anesthesia care) [21]. Therefore, we assumed that the decreasing of anxiety after surgery may be ascribed to the injection of DEX in this study. We will need to evaluate State-Trait Anxiety Inventory scale in a placebo group in further studies. But, patients’ anxiety and comfort level was not different regardless of the timing of sedation in our study. It might be single shot spinal anesthesia is a relatively easy and comfortable technique than epidural anesthesia or peripheral nerve block to perform.

The routine administration of DEX is limited because of the hypotension and bradycardia that may occur in the postoperative period [6, 22]. The time for recovery and discharge from the PACU was found to be longer with DEX than in the placebo group [21]. We also observed hypotension and bradycardia in the PACU. These side effects were reversible with the administration of fluid therapy or drugs, but anesthetists should beware of DEX infusion during the recovery time. The benefit of DEX as sedative is that it can cause minimal respiratory depression. However, we reported a relatively high incidence rate (30%) of desaturation during infusion of DEX. This may be because the definition of oxygen desaturation (SpO2 < 95%) in our study was more restricted than the definition applied in other studies (SpO2 < 90%) [14, 15].

There are some limitations to this study. First, we did not assess the differences regarding the duration of sensory or motor blockade produced by SA with DEX infusion. However, intravenous administration of DEX before or after SA has been found to prolong the duration of sensory and motor block in previous studies [12, 16, 23, 24]. Second, we excluded geriatric patients (>60 years) and those with hypertension and DM. Therefore, further studies are needed to investigate the efficacy of DEX in these patients.

## Conclusions

The intravenous administration of DEX before SA was induced more hypotension than the administration of DEX after SA. The level of anxiety and comfort of the patients was not different according to the timing of administration of DEX. The timing of infusion of DEX as sedative with SA is more preferable after performing of SA.

## Abbreviations

ASA: American society of anesthesiologists classification
BIS: Bispectral index
DEX: Dexmedetomidine
DM: Diabetes mellitus
ECG: Electrocardiography
HR: Heart rate
IQR: interquartile range
MAC: monitored anesthesia care
MBP: mean blood pressure
NIBP: non-invasive blood pressure
NRS: numerical rating scale
PACU: post-anesthetic care unit
PTSD: post-traumatic stress disorder
SA: spinal anesthesia
SD: standard deviation
STAI: Spielberger’s state-trait anxiety inventory
